# Structural connectivity differs between males and females in the brain object manipulation network

**DOI:** 10.1371/journal.pone.0253273

**Published:** 2021-06-11

**Authors:** Dongha Lee, Taekwon Son

**Affiliations:** 1 Cognitive Science Research Group, Korea Brain Research Institute, Daegu, Republic of Korea; 2 Korea Brain Bank, Korea Brain Research Institute, Daegu, Republic of Korea; Texas Tech University, UNITED STATES

## Abstract

Object control skills are one of the most important abilities in daily life. Knowledge of object manipulation is an essential factor in improving object control skills. Although males and females equally try to use object manipulation knowledge, their object control abilities often differ. To explain this difference, we investigated how structural brain networks in males and females are differentially organized in the tool-preferring areas of the object manipulation network. The structural connectivity between the primary motor and premotor regions and between the inferior parietal regions in males was significantly higher than that in females. However, females showed greater structural connectivity in various regions of the object manipulation network, including the paracentral lobule, inferior parietal regions, superior parietal cortices, MT+ complex and neighboring visual areas, and dorsal stream visual cortex. The global node strength found in the female parietal network was significantly higher than that in males but not for the entire object manipulation, ventral temporal, and motor networks. These findings indicated that the parietal network in females has greater inter-regional structural connectivity to retrieve manipulation knowledge than that in males. This study suggests that differential structural networks in males and females might influence object manipulation knowledge retrieval.

## Introduction

We manipulate objects (e.g., tools) in our daily life for various purposes. Object manipulation is the way we control objects (e.g., hammering action) [1]. Event-specific distinct properties of objects are invariant in situations, such as different exemplars of the same type of objects [2], different viewing conditions [3–5], and different object sizes [6–8]. Our knowledge of complex objects should be formed in the brain for stable object manipulation.

Many functional magnetic resonance imaging (fMRI) studies have shown functional activations in tool-related brain regions for object manipulation, including the premotor cortex [9–11], inferior parietal cortex [11–14], superior parietal cortex [14, 15], medial fusiform gyrus [12, 16], and ventral and dorsal stream areas [12, 13, 15, 17]. Object manipulation can also be represented by functional interactions between brain regions in the tool processing network [18], despite differential representations between visual features in the low-level visual space (e.g., orientation and edge) and high-level semantic space (e.g., tool and house) [19–22].

Spatial ability is required to selectively manipulate one object among many others. The spatial ability, including visuospatial skills [23] and visual processing [24], differs between sexes. Males show better spatial visualization, whereas females show better visual recognition [25, 26]. Besides, females have a substantial advantage over males in object processing, whereas males have an advantage over females in movement control [23]. To date, such sex differences and their underlying mechanisms remain controversial, despite accumulating evidence in favor of cognitive sex differences in brain structure [27–30], network [31], and sex hormones [32–34]. To our knowledge, it is not known why males and females manipulate objects differently, even though they use object manipulation knowledge.

A potential hypothesis to explain sex differences in object manipulation suggests that brain functions might vary if the structural connectivity is different. This hypothesis follows the idea of ‘Manifestation of the functionality from the structural network’ [35]. In other words, the structure-function relationships can explain the organizational principles of the brain system [36]. Thus, it is possible to detect the transfer of altered information to the structural network for functional network reorganization after anatomical damage [37]. By considering that the brain is structurally organized for global integration of local (segregated) functions [38, 39], sex differences in object manipulation could be explored in terms of structural brain networks. We expected to find differences in structural connections for object manipulation between males and females.

Here, we tested the hypothesis that structural connectivity for object manipulation knowledge retrieval differs between males and females. We constructed an object manipulation network of tool-preferring regions using a topic-based meta-analysis and cortical parcellation maps to test this hypothesis. The object manipulation network represented the brain regions involved in how tools are manipulated (e.g., how a hammer is used?). This approach allowed us to test how structural networks for object manipulation knowledge retrieval were differently manifested in males and females. Because the appropriate use of tools (e.g., cutting with a knife) requires the integration of action knowledge [11], retrieval of object manipulation knowledge is not restricted to one specific region but spreads over several regions [18]. The object manipulation network was divided into parietal, ventral temporal, and motor networks to test subsystem specificity.

Structural connectivity was calculated in the object manipulation network and its subnetworks using probabilistic tractography. The structural network properties (global node strength and global efficiency) were based on theoretical graph measures. These were evaluated to investigate sex differences by assuming that structural connectivity changes could be reliably detected in many edges rather than at a single edge level [16, 40]. Previous studies [41–43] suggested that grip strength was related to the physical consequences of object manipulation. Therefore, we tested whether males and females showed different abilities to control objects through behavioral motor scores and how their ability was related to the structural network properties in the object manipulation network.

## Materials and methods

### Data acquisition

Diffusion tensor imaging (DTI) data and T1-weighted MRI scans of 100 participants were randomly obtained from the Human Connectome Project (HCP) database [44]. These were preprocessed data in the HCP 1200 Subjects release. After completing the DTI analysis, we found that our dataset included 41 males (mean age = 28.9, SD = 3.7, range = 22–35 years) and 59 females (mean age = 30.1, SD = 3.4, range = 24–36 years). We excluded 18 females to achieve an equal number of males and females and match them for age (new mean age = 28.9, SD = 3.1, range = 24–36 years). Detailed information on the MRI sequences is available from the HCP 1200 Subjects release image and behavioral data (https://www.humanconnectome.org/study/hcp-young-adult/document/1200-subjects-data-release).

### Object manipulation network

**Fig 1:**
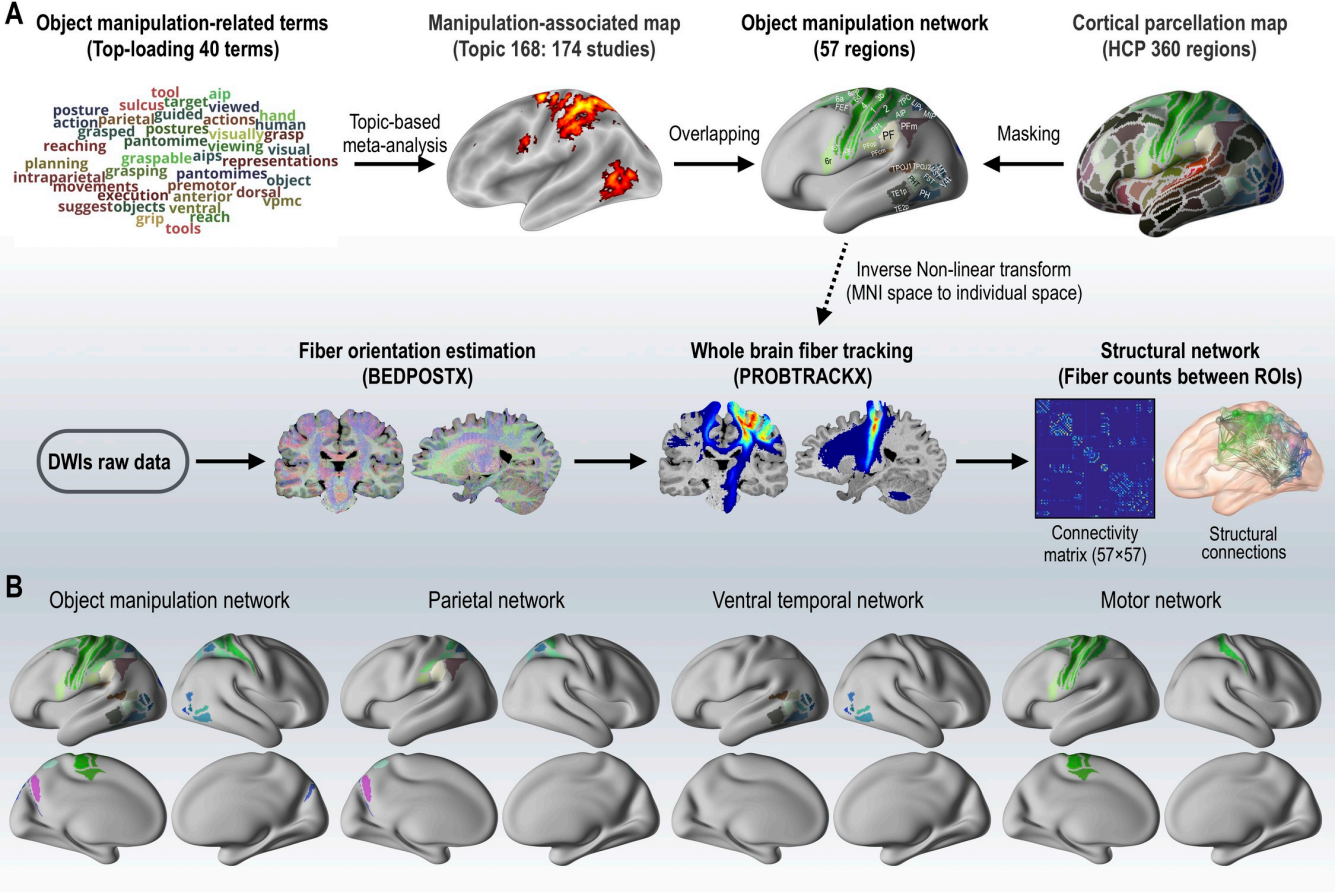
Data selection and processing. (A) An overview of the meta-analysis procedure to derive the object manipulation network and its structural network construction. FEF, frontal eye fields; 6a, area 6 anterior; 6mp, area 6mp (supplementary motor area); 6d, dorsal area 6; 6r, rostral area 6; 6v, ventral premotor cortex 6; 4, primary motor cortex; 3a, area 3a; 3b, primary sensory cortex; 1, area 1; 2, area 2; PFt, PFt area; Pfop, PF opercular area; PF, PF area (Brodmann Area 40); PFm, PF Complex area; 7PC, Area 7PC; AIP, anterior intraparietal area; LIPv, ventral lateral intraparietal area; MIP, medial intraparietal Area; TPOJ1, temporo-parieto-occipital junction 1 area; TPOJ2, temporo-parieto-occipital junction 2 area; TE1p, posterior TE1 area; TE2p, posterior TE2 area; PHT, PHT area; PH, PH area; FST, FST area; MST, medial superior temporal Area; MT, middle temporal Area; V4t, V4t area. (B) Construction of the object manipulation network and its subnetwork regions. L, left; R, right; BEDPOSTX, Bayesian estimation of diffusion parameters obtained using sampling techniques; PROBTRACKX, probabilistic tracking with crossing fibers.

We used a functional map representing object manipulation associations and a cortical parcellation map comprising 360 cortical regions from the Montreal Neurological Institute template space to construct an object manipulation network. We selected topic 168 as a functional map from a set of 400 topics in the Neurosynth database [45] through a topic-based meta-analysis of the activation coordinates reported in 174 studies. Object manipulation-related terms, such as ‘tool,’ ‘object,’ and ‘hand’ were well represented in topic 168. More details of the topic-based meta-analysis can be found in the study by Poldrack et al. [46]. The terms used for the topic-based meta-analysis are summarized in S1A Fig. The cortical parcellation map was based on an HCP’s multi-modal parcellation atlas that comprises 360 cortical (gray matter) regions [47]. We combined the functional and cortical maps to construct an object manipulation network. By overlapping the maps and using a threshold of at least 100 contiguous voxels, we retained 57 cortical regions. The procedures conducted in this study are presented in Fig 1A, and the object manipulation network map is displayed in S1B Fig.

Parietal, ventral temporal, and motor networks were constructed to investigate topological properties in the object manipulation network and its subnetworks (Fig 1B). The parietal network consisted of 22 regions in the inferior parietal cortex (L.PFt, L.Pfop, L.PF, L.PFm, and R.IP2), superior parietal cortex (L.7AL, R.7AL, L.7Am, R.7PL, L.7PC, R.7PC, L.LIPv, R.LIPv, L.VIP, R.VIP, L.MIP, R.MIP, R.LIPd, L.AIP, and R.AIP), and posterior cingulate cortex (L.POS2 and L.DVT) [48–50]. The ventral temporal network was composed of 15 regions in the lateral temporal cortex (L.TE1p, R.TE1p, and L.PHT), temporo-parieto-occipital junction (L.TPOJ1 and L.TPOJ2), and MT+ complex and neighboring visual areas (L.MST, R.LO2, L.MT, L.PH, R.PH, L.V4t, R.V4t, L.FST, R.FST, and R.LO3) [51–53]. The motor network included 14 regions in the premotor cortex (L.FEF, L.6d, L.6v, L.6r, and L.6a), paracentral lobular and mid-cingulate cortex (L.24dd, L.6ma, and L.6mp), and somatosensory and motor cortex (L.4, L.3b, L.1, L.2, R.2, and L.3a) [54–58].

### Data processing

Probabilistic tractography was conducted in the individual DTI space (native space). T1-weighted MRI scans were sampled from the HCP database to have the same resolution as the DTI data. The object manipulation network map in the Montreal Neurological Institute template space was transformed into the individual T1-weighted MRI scans by applying the inverse deformation field in the DARTEL algorithm in SPM8 [59]. This led to the inverse non-linear transformation from individual T1-weighted MRI scans to the International Consortium for Brain Mapping (ICBM) T1-weighted MRI template. After the transformation, the 57 nodes of the object manipulation network map were defined in individual DTI spaces. The nodes were used as seed masks to calculate a connectivity distribution between them. The transformation processes are summarized in S2 Fig.

### Structural network construction

We carried out a probabilistic tractography on individual DTI datasets to calculate structural connectivity between brain regions of the object manipulation network. The DTI data were preprocessed using FMRIB’s diffusion toolbox (FDT v3.0, http://fsl.fmrib.ox.ac.uk/fsl/fslwiki/FDT) as far as the Bayesian estimation of diffusion parameters obtained using sampling techniques (BEDPOSTX), following the HCP Diffusion pipeline [60, 61]. We conducted probabilistic tracking with crossing fibers (PROBTRACKX) to generate probabilistic connections using the preprocessed DTI data. The probabilistic tracking parameters were as follows: 5,000 samples within each voxel, 0.2 curvature threshold, 0.5-mm step length, and 2,000 steps per sample. The structural connectivity between tool-preferring regions was calculated by the number of probabilistic streamlines projecting from one tool-preferring region to another. Since the brain region sizes differ between males and females [62], presumably leading to differences in the structural connectivity, the probabilistic streamlines were normalized by the size of the seed region of interest. The normalized probabilistic streamlines were used to construct a structural matrix for each participant, without thresholding for the object manipulation network.

### Analysis of the object manipulation network

We performed theoretical graph analysis using the structural connectivity matrix and BCT toolbox of MATLAB to investigate the global topological properties of the object manipulation network and its three subnetworks in males and females [63] (MathWorks, Inc.).

The strength of the *i*-th node was the sum of all connection weights between it and the other nodes. Edge strength was defined as the structural connectivity at each edge. Two-sample *t*-tests were used to evaluate sex differences in the local node properties. Statistical differences were considered significant at a false discovery rate of 0.05.

The global node strength, *S_gl_*, was calculated as the average strength of all nodes, using the following formula:

$$S_{gl}=\frac{1}{N}\sum_{j\in G}w_{i,j}$$

where *w* is the correlation coefficients between the *i*-th and *j*-th nodes. *N* indicates the total number of nodes, and *G* is the adjacency matrix of the structural network.

Node efficiency was defined as the mean of all shortest path length pairs. Global efficiency, *E_gl_*, is the average of all node efficiencies [64], defined as the inverse of the path length (the fewest number of edges between the *i*-th and *j*-th nodes):

$$E_{gl}=\frac{1}{N(N-1)}\sum_{i\neq j}\sum_{j\in G,j\neq i}{{(d}_{i,j})}^{-1}$$

where *d* is the geodesic path between the *i*-th and *j*-th nodes.

Two-sample *t*-tests were used to investigate sex differences in the global node strength and global efficiency. Significant differences between brain networks were assessed following Bonferroni correction for multiple comparisons.

## Relationship between global properties and behavioral motor scores

This study aimed to investigate whether behavioral motor scores related to object manipulation (e.g., grip strength and dexterity in the National Institutes of Health toolbox [65]) could explain differences in structural connectivity between males and females. For grip strength testing, males and females squeezed the dynamometer that was used to measure the force in pounds, while counting to three. For dexterity testing, males and females placed nine plastic pegs into a pegboard and removed them as accurately as they could. To this end, we evaluated the association between the structural connectivity global properties in the object manipulation network and its subnetworks and the grip strength as well as between the structural global properties and dexterity testing scores. We used the Pearson correlation coefficient to examine the relationships of global node strength with grip strength and dexterity and of global efficiency with grip strength and dexterity.

## Results

### Differences in structural connectivity between males and females

**Fig 2:**
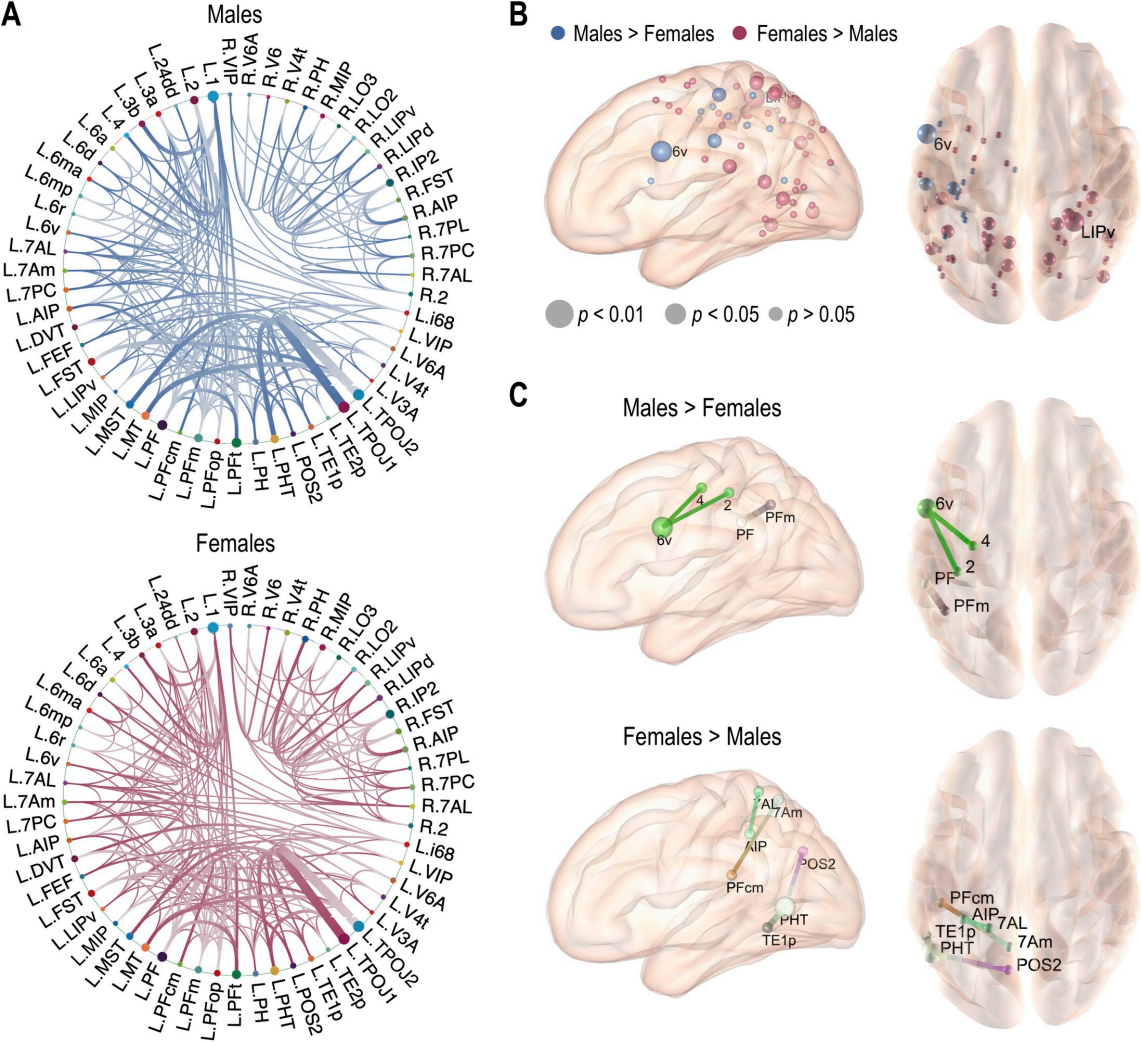
Structural connectivity differences between males and females. (A) Structural connectivity in the object manipulation network in males and females. Significant differences in node strength (B) and edge strength (C) between males and females (two-sample *t*-tests).

We compared global properties of the object manipulation network to investigate sex differences in structural connectivity. Fig 2 shows the structural connectivity in the object manipulation network in males and females. Group-average connections are displayed as blue lines for males and red lines for females (Fig 2A). As shown in Fig 2B, node strengths in the left motor-related regions were higher in males than females, whereas in the parietal and temporal regions, they were higher in females than males. Edge strengths of the left ventral premotor cortex (6v) with the primary motor cortex (4) and S1 primary somatosensory complex (2) and between the left inferior parietal cortical regions (PF and PFm) were significantly higher in males than females. However, edge strengths between the paracentral lobule, inferior and superior parietal cortices, middle temporal complex (MT+) and neighboring visual areas, and dorsal stream visual cortex were significantly higher in females than males (Fig 2C). The statistical group differences are summarized in S1 and S2 Tables.

### Sex differences in the structural network for object manipulation

**Fig 3:**
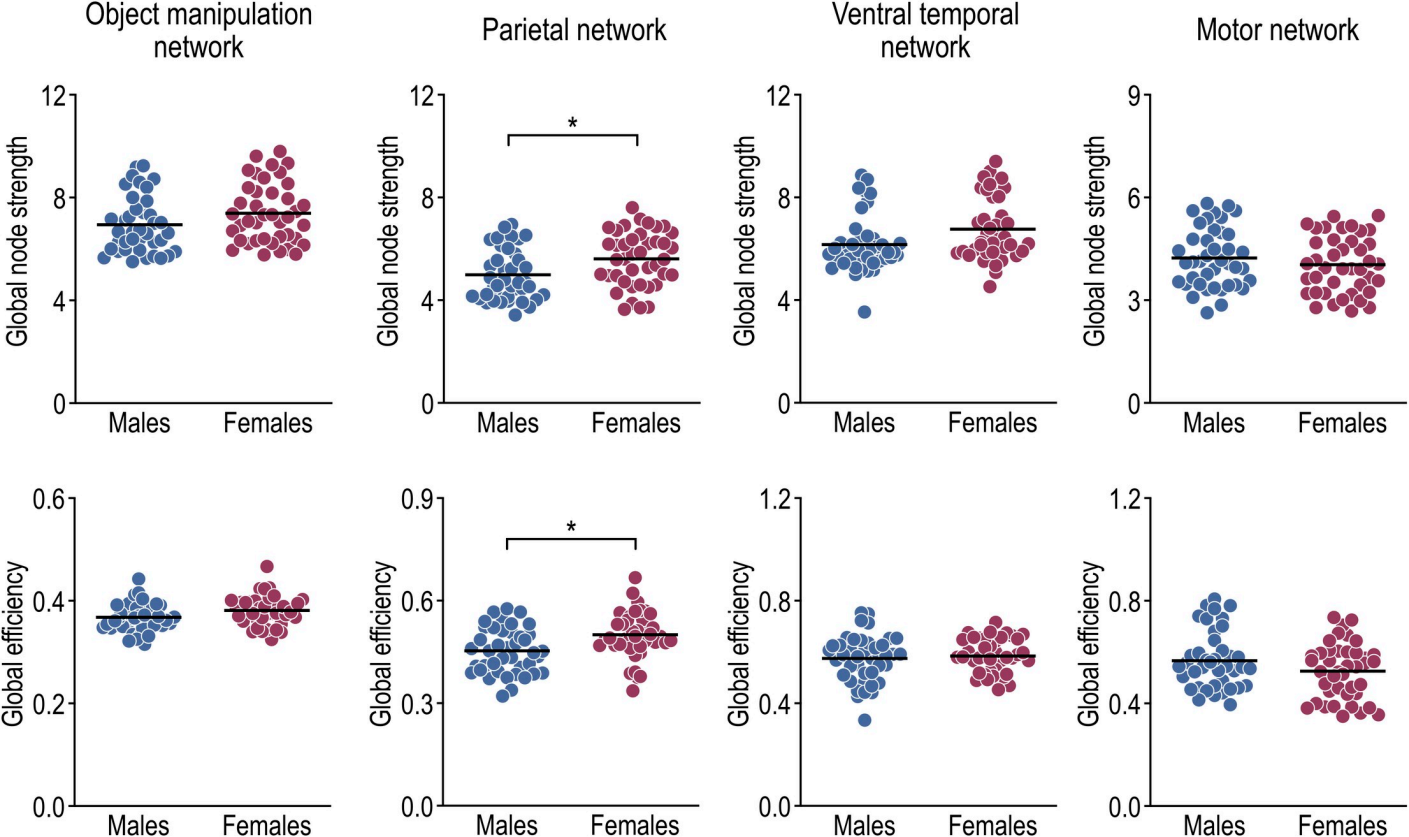
Global node strength and global efficiency of structural networks in the object manipulation, parietal, ventral temporal, and motor networks. *p < 0.05.

Fig 3 displays the global network properties of the entire object manipulation network and those of the three subnetworks. Statistical significance in the network analysis was defined as a two-sided *p*-value < 0.05, and a Bonferroni correction for multiple comparisons was used for the entire object manipulation network and its subnetworks (adjusted significance level: *p* < 0.0125). The global node strength and global efficiency for the entire object manipulation network were similar between males and females (two-sample *t*-tests; mean ± standard error of the mean of global node strength, males = 6.94 ± 1.07 vs. females = 7.39 ± 1.17, t(80) = 1.82, *p* = 0.072; global efficiency, males = 0.37 ± 0.03 vs. females = 0.38 ± 0.03, t(80) = 2.20, *p* = 0.031). The parietal network global node strength and global efficiency in females were significantly higher than those in males (global node strength: males = 4.99 ± 1.02 vs. females = 5.61 ± 1.05, t(80) = 2.72, *p* = 0.008; global efficiency: males = 0.45 ± 0.07 vs. females = 0.50 ± 0.07, t(80) = 3.18, *p* = 0.002). However, males and females were similar in global node strength and global efficiency in the ventral temporal network (global node strength: males = 6.16 ± 1.11 vs. females = 6.76 ± 1.24, t(80) = 2.30, *p* = 0.024; global efficiency: males = 0.57 ± 0.09 vs. females = 0.58 ± 0.07, t(80) = 0.54, *p* = 0.588). Furthermore, we found no sex differences in the global properties of the motor network (global node strength: males = 4.23 ± 0.87 vs. females = 4.04 ± 0.84, t(80) = 1.04, *p* = 0.301; global efficiency: males = 0.57 ± 0.11 vs. females = 0.53± 0.11, t(80) = 1.66, *p* = 0.101).

**Fig 4:**
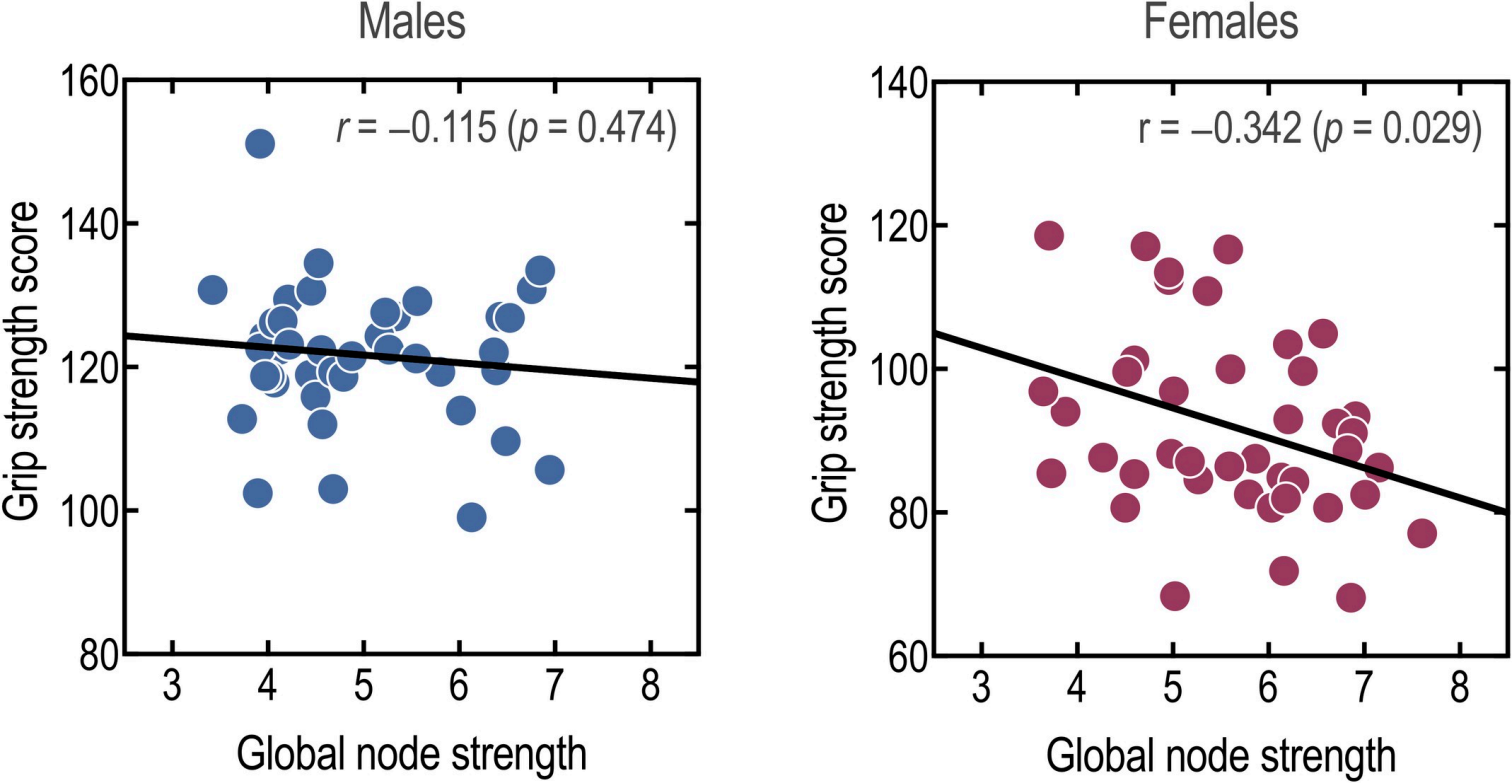
Correlation analysis between the parietal network global node strength and grip strength in males and females.

Males showed significantly higher grip strength than females (males = 121.53 ± 1.50 vs. females = 91.87 ± 2.01, t(80) = 11.84, *p* < 0.001), while females showed significantly higher dexterity than males (males = 110.57 ± 1.03 vs. females = 115.85 ± 1.67, t(80) = 8.89, *p* = 0.007). Fig 4 shows the relationships between the global node strength in the parietal network and behavioral motor scores derived from the grip strength (a measure of muscle capacity to control movement). Intriguingly, the global node strength was negatively correlated to the grip strength score in females (*r* = −0.342, *p* = 0.029) but not in males (*r* = −0.115, *p* = 0.474). Furthermore, there was no association between the global efficiency and grip strength in males or females (males: *r* = 0.03, *p* = 0.866; females: *r* = −0.26, *p* = 0.096). By contrast, the global node strength tended insignificantly to positively correlate with the dexterity score, a measure of the ability to manipulate objects in a timely manner, in females but not males (males: *r* = 0.179, *p* = 0.262; females: *r* = 0.286, *p* = 0.070; S3 Fig).

## Discussion

This study aimed to investigate differences between males and females in structural connectivity to retrieve object manipulation knowledge. The structural network properties (global node strength and global efficiency) of males and females were assessed in the object manipulation network and its subnetworks. The global node strength and global efficiency in the parietal network were higher in females than males but not in the entire object manipulation, ventral temporal, and motor networks. This observation suggests that the parietal network has greater inter-regional structural connectivity for manipulation knowledge retrieval in females than males.

The present study showed sex differences in structural connectivity between tool-preferring regions. Specifically, the nodes with high connection strengths were primarily distributed in the parietal regions of females. The edge strengths between the left motor-related regions and between the left inferior parietal regions were higher in males than females, while the edge strengths between various tool-preferring regions were higher in females than males. These findings support the sex-dependent differential structural organization of the object manipulation network. Females had highly interconnected nodes as hubs, especially in the posterior parietal cortex, while males did not. Moreover, males had sparse connections in the parietal network, whereas these were dense in females. The parietal regions did not work in isolation in females but rather interacted with other regions. This interaction probably expanded tool-specific information in modules (for local segregation) to the tool-preferring network (for global integration), as detailed in several reviews on brain organization [36, 38, 66].

Concerning topographical organization, the structural connectivity difference between males and females was parietal network-specific. The higher global node strength and global efficiency in females were observed in the parietal network but not in the ventral temporal and motor networks. Higher global node strength and global efficiency indicated increased inter-regional structural connectivity. This suggested that females have highly interconnected nodes and dense connections in the parietal network. Despite the modality difference, our findings accorded with previous observations, thus demonstrating a pivotal role for the parietal regions, including the inferior parietal lobule [11, 12, 14], superior parietal cortex [14, 15], and supramarginal gyrus [16, 67], in object action processing. Therefore, it is necessary to consider the sex effect on object manipulation when targeting the parietal regions as structural connectivity changes might affect functional connectivity. Using sex as a confound regressor could improve accuracy when assessing neural activity or functional connectivity for object representation based on fMRI.

This study demonstrated that the parietal network shows sex-specific structural connectivity. We then asked how this structural connectivity difference was related to the dissimilar behavioral or physical ability to control objects between males and females. According to previous studies [12–14, 68], this difference is because the parietal network is involved in the spatial ability to manipulate objects. Grip strength-dependent changes in structural connectivity were found in the parietal network of females but not males. The global node strength of the parietal network in females was inversely correlated with the grip strength (Fig 4). This finding is consistent with previous studies, demonstrating the involvement of the parietal cortex in grip control with regard to object manipulation [69, 70].

It is assumed that sex differences for object manipulation in the parietal network reflect differences in physical and behavioral assessment between the sexes. Males showed higher grip strength, while females showed higher dexterity. These sex differences indicate that females’ structural network for manipulation knowledge retrieval was more efficient than motor representations in the parietal cortex [71–73]. Indeed, our results demonstrated that the stronger the structural connectivity in the parietal network, the weaker the grip strength and the higher the dexterity performance.

This study was limited to young adults (aged 22–36 years) as the HCP database collected MRI data from 1,200 individuals of this age group only. The applicability of our results on the differences between the sexes to younger and older populations is unknown. Future studies will need to investigate the reliability of sex differences in structural connectivity in children and older adults. An additional limitation may be the lack of functional information related to the cause of such sex differences. As the current study was only performed on one population, more studies are necessary to clarify whether there are genetic differences that result in such differences in connectivity or are the differences in connectivity due to neuroplasticity and learned skills during early development.

In addition, there is a methodological limitation in using streamline count, regardless of whether it is derived from deterministic or probabilistic tractography. This is because streamline count cannot explain the quantitative nature of whole-brain streamline reconstructions and measure the reliable mapping between axon pathways and diffusion profile [74–76]. Since using streamline count to reconstruct the structural network is fundamentally problematic for structural network analyses owing to biases introduced into the tractogram [77], it should be carefully interpreted. To address these issues, one of the tractogram post-processing techniques, spherical-deconvolution informed filtering of tractograms [78], has been proposed and demonstrated to improve the biological accuracy [79]. However, since we used probabilistic streamline tractography that needs fiber orientation estimates and estimates of their uncertainty to reconstruct a dominant probability path to a seed, we could not apply the tractogram filtering technique based on spherical-deconvolution-based tractography. Further studies based on probabilistic streamline tractography should attempt to overcome these limitations.

Collectively, our results demonstrated sex differences in the structural network for object manipulation knowledge retrieval. These findings suggested that females have a more efficient structural organization for object manipulation knowledge retrieval than males. Specifically, the parietal network was particularly sex-sensitive for assessing object manipulation knowledge.

## Supporting information

S1 Fig Object manipulation functional map and network cortical regions. (A) An object manipulation functional map. The map was constructed based on topic 168 (total 174 studies) in a set of 400 topics from the meta-analytic Neurosynth database of 14,371 published fMRI studies). Top-loading terms for topic 168 (https://www.neurosynth.org/analyses/topics/v5-topics-400/168) were tool, object, hand, grasping, tools, reaching, grasp, intraparietal, action, actions, objects, reach, sulcus, anterior, premotor, parietal, aips, planning, movements, ventral, viewing, guided, aip, grip, visual, human, dorsal, visually, target, suggest, pantomimes, grasped, graspable, pantomime, viewed, postures, execution, vpmc, posture, representations. (B) The object manipulation network comprised 57 cortical regions.
(TIF)

S2 Fig General method for transforming images from Montreal Neurological Institute (MNI) space to the individual native space. The statistical parametric mapping (SPM) saves the forward and inverse deformation fields in SPM segmentation. The images in the MNI space can be transformed into the individual diffusion tensor imaging (DTI) space using the inverse deformation fields in SPM normalization.
(TIF)

S3 Fig Correlation analysis between the parietal network global node strength and dexterity in males and females. (TIF)

S1 Table Sex differences in node strengths. L: left, R: right, SD: standard deviation, *: false discovery rate (FDR) < 0.05.
(PDF)

S2 Table Sex differences in edge strengths. Reduced edges with a false discovery rate (FDR) < 0.02 were observed in the motor network of females, but not in males. In contrast, increased edges were observed in the parietal network of females compared to males. L: left, R: right, SD: standard deviation.
(PDF)
