# Supplementary figures and images for: Structural connectivity differs between males and females in the brain object manipulation network
Source: PLoS One. 2021 Jun 11;16(6):e0253273. doi: 10.1371/journal.pone.0253273 (PMC8195422; doi:10.1371/journal.pone.0253273)

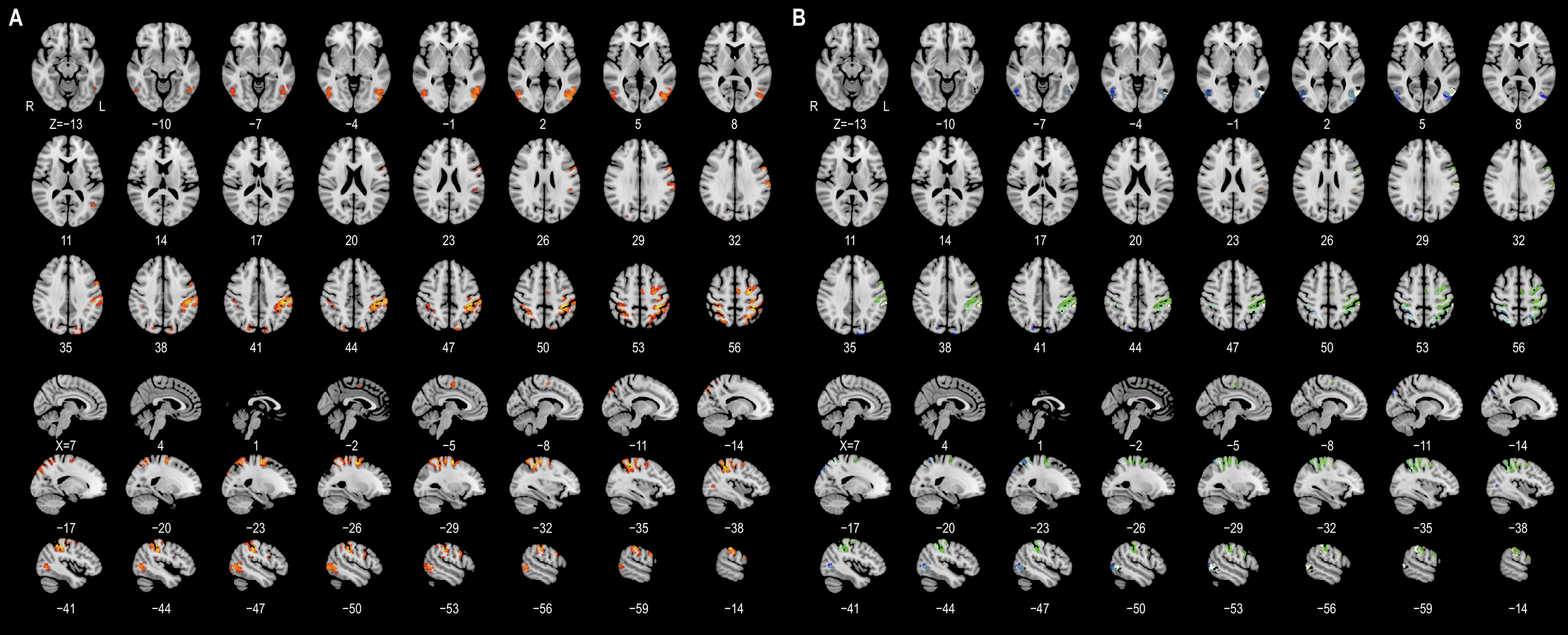

Supplement: S1 Fig — (A) An object manipulation functional map. The map was constructed based on topic 168 (total 174 studies) in a set of 400 topics from the meta-analytic Neurosynth database of 14,371 published fMRI studies). Top-loading terms for topic 168 (https://www.neurosynth.org/analyses/topics/v5-topics-400/168) were tool, object, hand, grasping, tools, reaching, grasp, intraparietal, action, actions, objects, reach, sulcus, anterior, premotor, parietal, aips, planning, movements, ventral, viewing, guided, aip, grip, visual, human, dorsal, visually, target, suggest, pantomimes, grasped, graspable, pantomime, viewed, postures, execution, vpmc, posture, representations. (B) The object manipulation network comprised 57 cortical regions. (TIF) [file pone.0253273.s001.tif]

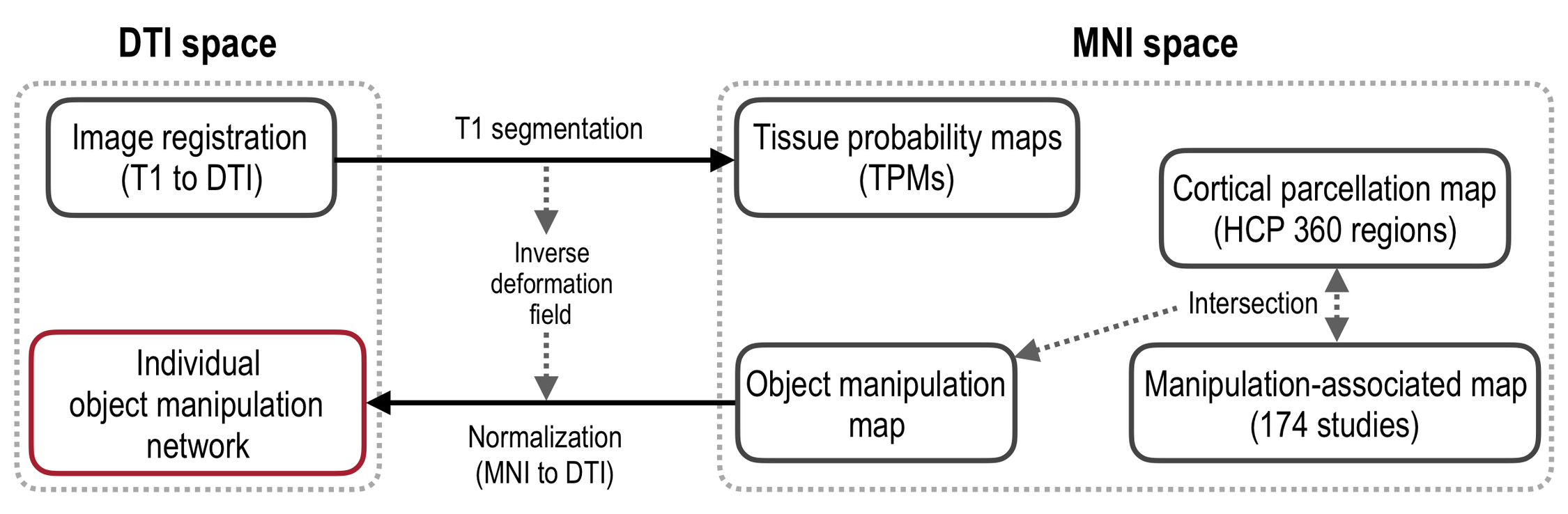

Supplement: S2 Fig — The statistical parametric mapping (SPM) saves the forward and inverse deformation fields in SPM segmentation. The images in the MNI space can be transformed into the individual diffusion tensor imaging (DTI) space using the inverse deformation fields in SPM normalization. (TIF) [file pone.0253273.s002.tif]

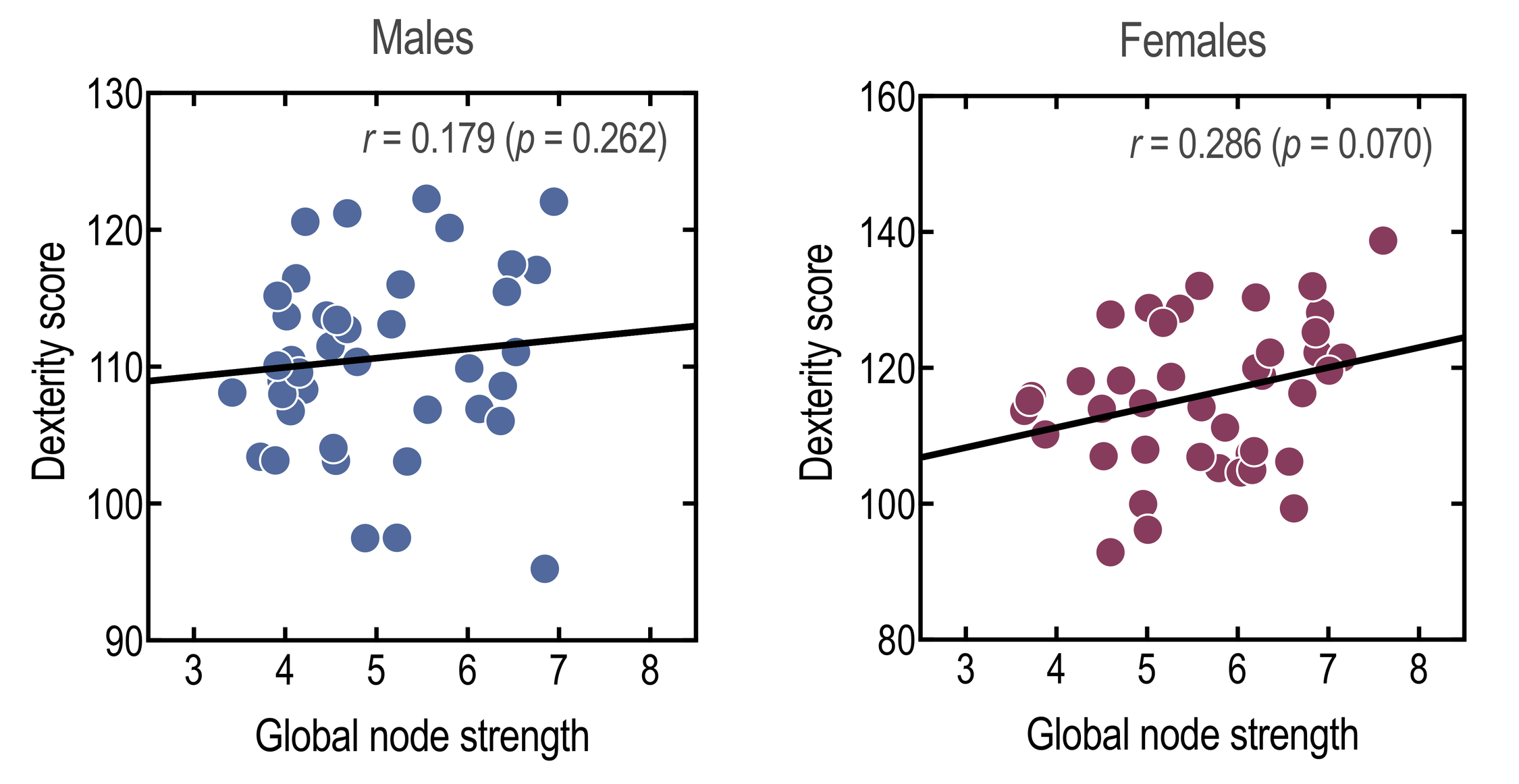

Supplement: S3 Fig — (TIF) [file pone.0253273.s003.tif]
