# Supplementary material for: Structural connectivity differs between males and females in the brain object manipulation network
Source: PLoS One. 2021 Jun 11;16(6):e0253273. doi: 10.1371/journal.pone.0253273 (PMC8195422; doi:10.1371/journal.pone.0253273)
Supplement: S1 Table — L: left, R: right, SD: standard deviation, *: false discovery rate (FDR) < 0.05. (PDF) [file pone.0253273.s004.pdf]

HCP area	Cortical pacellation region	Men		Women		Difference
		Mean	SD	Mean	SD	p-value
L.4	Somatosensory and Motor Cortex	5.49	1.68	5.22	1.58	0.4667
L.3b		6.04	1.30	5.75	1.38	0.3391
L.1		22.06	9.97	16.80	11.12	0.0270*
L.2		11.33	2.04	10.66	2.07	0.1424
R.2		4.75	1.90	5.52	2.15	0.0875
L.3a		5.86	2.18	6.50	3.44	0.3192
L.24dd	Paracentral Lobular and Mid Cingulate Cortex	2.38	2.47	2.08	0.99	0.4751
L.6ma		7.41	2.99	8.22	2.38	0.1777
L.6mp		4.40	1.87	4.88	1.62	0.2186
L.FEF	Premotor Cortex	5.98	2.00	6.38	2.28	0.4104
L.6d		5.90	1.38	6.31	1.49	0.2039
L.6v		7.37	2.29	5.97	2.36	0.0076*
L.6r		5.08	1.46	5.00	2.02	0.8433
L.6a		6.13	1.28	6.46	1.38	0.2700
L.MST	MT+ Complex and Neighboring Visual Areas	7.55	2.05	8.45	2.35	0.0670
R.LO2		4.53	1.97	5.51	1.99	0.0280*
L.MT		9.45	2.87	10.47	3.40	0.1439
L.PH		8.91	1.93	9.56	1.92	0.1322
R.PH		4.27	1.29	5.85	3.74	0.0126*
L.V4t		5.79	2.41	6.72	2.74	0.1041
R.V4t		4.31	1.54	4.95	1.62	0.0707
L.FST		8.30	2.26	9.18	2.39	0.0914
R.FST		4.41	1.33	4.74	1.55	0.2981
R.LO3		3.15	2.34	4.11	2.91	0.1068
L.TE1p	Lateral Temporal Cortex	8.88	2.02	9.38	2.58	0.3383
L.TE2p		4.88	2.56	5.48	2.51	0.2889
L.PHT		9.18	2.11	10.47	2.55	0.0146*
L.TPOJ1	Temporo-Parieto-Occipital Junction	6.71	4.69	9.59	7.05	0.0326*
L.TPOJ2		19.39	10.46	16.23	8.75	0.1416
L.7AL	Superior Parietal Cortex	5.71	1.59	6.89	2.48	0.0120*
R.7AL		6.68	2.42	8.86	4.98	0.0140*
L.7Am		8.79	3.37	10.67	4.09	0.0263*

R.7PL		2.84	1.91	3.96	2.33	0.0199*
L.7PC		8.16	1.48	8.06	1.73	0.7973
R.7PC		7.32	1.89	7.76	1.81	0.2828
R.LIPd		4.28	2.63	5.80	2.90	0.0147*
L.AIP		7.24	1.37	7.20	2.13	0.9307
R.AIP		4.68	1.77	5.56	1.72	0.0250*
L.LIPv	Ventral visual stream network	6.16	2.04	6.36	2.27	0.6790
R.LIPv		5.33	1.96	6.59	2.28	0.0093*
L.VIP		5.69	1.49	6.49	1.72	0.0265*
R.VIP		5.66	1.63	6.49	2.25	0.0582
L.MIP		4.42	1.96	6.17	5.98	0.0795
R.MIP		5.32	4.77	7.61	6.53	0.0727
L.PFt	Inferior Parietal Cortex	12.68	3.78	10.48	4.94	0.0262*
R.IP2		8.35	2.98	8.38	4.18	0.9655
L.PFop		6.59	3.08	7.70	3.94	0.1605
L.PF		12.98	7.70	13.38	7.58	0.8109
L.PFm		17.47	7.32	15.08	7.24	0.1411
L.POS2	Posterior Cingulate Cortex	7.53	3.73	9.19	3.80	0.0500
L.DVT		6.46	2.09	6.91	3.87	0.5077
L.V3A	Early Visual Cortex	4.12	2.21	4.87	2.14	0.1213
R.V6	Dorsal Stream Visual Cortex	2.37	1.64	2.95	1.71	0.1195
L.V6A		5.04	1.83	5.80	1.94	0.0700
R.V6A		3.53	2.51	4.38	2.36	0.1184
L.PFcm	Posterior Opercular Cortex	2.85	1.48	3.59	1.65	0.0364*
L.i6_8	DorsoLateral Prefrontal Cortex	7.45	3.42	7.73	4.26	0.7407
