# Supplementary material for: Structural connectivity differs between males and females in the brain object manipulation network
Source: PLoS One. 2021 Jun 11;16(6):e0253273. doi: 10.1371/journal.pone.0253273 (PMC8195422; doi:10.1371/journal.pone.0253273)
Supplement: S2 Table — Reduced edges with a false discovery rate (FDR) < 0.02 were observed in the motor network of females, but not in males. In contrast, increased edges were observed in the parietal network of females compared to males. L: left, R: right, SD: standard deviation. (PDF) [file pone.0253273.s005.pdf]

Edge (region to region)	Men		Women		Difference
	Mean	SD	Mean	SD	p-value
Males > Females					
L.4 – L.6v	2.10	1.36	1.20	0.84	0.0005
L.2 – L.6v	1.17	1.07	0.63	0.66	0.0078
L.PF – L.PFm	40.15	31.37	22.93	19.64	0.0038
Females > Males					
L.7AL – L.AIP	0.02	0.16	0.39	0.70	0.0017
L.7Am – L.PFcm	0.37	0.70	0.85	0.88	0.0068
L.TE1p – L.PHT	12.95	6.41	18.10	9.67	0.0057
L.POS2 – L.PHT	0.12	0.40	0.61	0.77	0.0006
